# Radiomics-Based Analysis of Intestinal Ultrasound Images for Inflammatory Bowel Disease: A Feasibility Study

**DOI:** 10.1093/crocol/otae034

**Published:** 2024-05-16

**Authors:** Phillip Gu, Jui-Hsuan Chang, Dan Carter, Dermot P B McGovern, Jason Moore, Paul Wang, Xiuzhen Huang

**Affiliations:** F. Widjaja Inflammatory Bowel Disease Institute, Cedars-Sinai Medical Center, Los Angeles, CA, USA; Department of Computational Biomedicine, Cedars-Sinai Medical Center, Los Angeles, CA, USA; Department of Gastroenterology, Sheba Medical Center, Tel Aviv, Israel; F. Widjaja Inflammatory Bowel Disease Institute, Cedars-Sinai Medical Center, Los Angeles, CA, USA; Department of Computational Biomedicine, Cedars-Sinai Medical Center, Los Angeles, CA, USA; Department of Computational Biomedicine, Cedars-Sinai Medical Center, Los Angeles, CA, USA; Department of Computational Biomedicine, Cedars-Sinai Medical Center, Los Angeles, CA, USA

**Keywords:** inflammatory bowel disease, artificial intelligence, radiomics, convolutional neural network, intestinal ultrasound

## Abstract

**Background:**

The increasing adoption of intestinal ultrasound (**IUS**) for monitoring inflammatory bowel diseases (**IBD**) by IBD providers has uncovered new challenges regarding standardized image interpretation and limitations as a research tool. Artificial intelligence approaches can help address these challenges. We aim to determine the feasibility of radiomic analysis of IUS images and to determine if a radiomics-based classification model can accurately differentiate between normal and abnormal IUS images. We will also compare the radiomic-based model’s performance to a convolutional neural network (**CNN**)-based classification model to understand which method is more effective for extracting meaningful information from IUS images.

**Methods:**

Retrospectively analyzing IUS images obtained during routine outpatient visits, we developed and tested radiomic-based and CNN-based models to distinguish between normal and abnormal images, with abnormal images defined as bowel wall thickness > 3 mm or bowel hyperemia with modified Limberg score ≥ 1 (both are surrogate markers for inflammation). Model performances were measured by area under the receiver operator curve (**AUC**).

**Results:**

For this feasibility study, 125 images (33% abnormal) were analyzed. A radiomic-based model using XG boost yielded the best classifier model with average test AUC 0.98%, 93.8% sensitivity, 93.8% specificity, and 93.7% accuracy. The CNN-based classification model yielded an average testing AUC of 0.75.

**Conclusions:**

Radiomic analysis of IUS images is feasible, and a radiomic-based classification model could accurately differentiate abnormal from normal images. Our findings establish methods to facilitate future radiomic-based IUS studies that can help standardize image interpretation and expand IUS research capabilities.

## Graphical Abstract

**Graphical Abstract:**
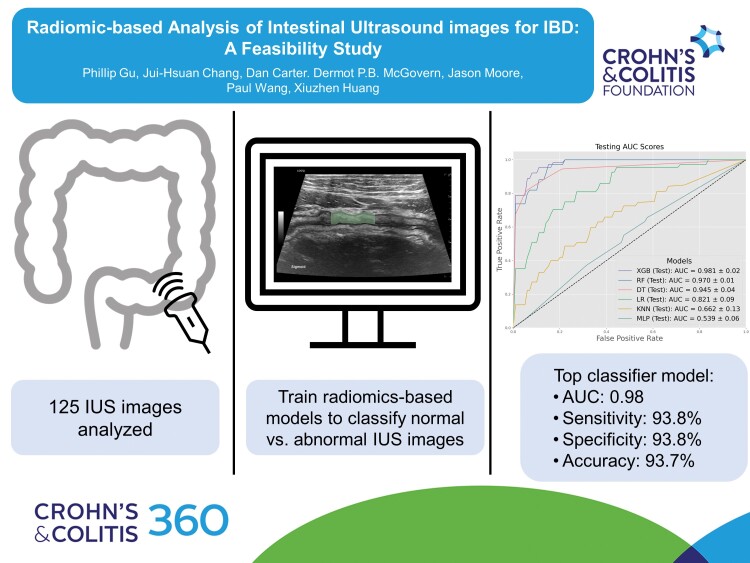

## Introduction

The evolution of ultrasound technology has facilitated the emergence of intestinal ultrasound (**IUS**) as a valuable, noninvasive, point-of-care tool for monitoring inflammatory bowel diseases (**IBD**) thereby helping IBD providers make real-time decisions at the bedside. IUS has excellent sensitivity and specificity for detecting inflammation and is a promising research tool for clinical trials and biomarker discovery.^1^ However, the increased adoption of IUS for IBD has uncovered new challenges. First, the growing interest among IBD providers to perform IUS in their practice has led to an increase in novice operators. Learning IUS can be challenging, and novice IUS users must overcome a learning curve before achieving basic competence.^2^ Considering many IUS parameters for inflammation are, at best, semi-quantitative (except for bowel wall thickness [BWT]), there is an increased risk of diagnostic errors stemming from IUS image interpretation by inexperienced operators. This has created a need to support less experienced IUS operators to ensure standardized and accurate image interpretation. Second, IUS is an ideal research tool for imaging biomarker discovery because it is noninvasive and radiation-sparing, but current approaches for biomarker discovery with IUS are confined to parameters defined a priori by human expert consensus.^3^ This approach may inadvertently overlook important parameters that are not readily detected by the human eye that could improve biomarker discovery and potentially yield additional insight into the biological underpinnings of IBD.

Artificial intelligence (**AI**) may offer solutions to address the current challenges in IUS. For AI-based medical imaging analysis in IBD, convolutional neural networks (**CNN**) and radiomics are the most used.^4^ CNN is an artificial neural network that uses images as input and can perform automated tasks such as image classification, object detection, segmentation, and image generation by automatically learning to identify the most predictive features directly from the image through a series of convolutional and pooling layers. However, with a CNN model, there exists a “black box” wherein the process through which the model arrives at its decisions and prediction is unknown, rendering CNNs difficult to interpret. This inherent limitation of CNN has been controversial for medical applications of CNN when trust and transparency are critical. On the other hand, radiomics is an objective and quantitative approach to analyze medical imaging through the mathematical extraction of specific spatial distribution of signal intensities and pixel interrelationships.^5^ Because extraction of specific features is required, radiomics-based models are more interpretable and can be integrated with other data types, such as transcriptomics or genomics, to develop multidimensional prediction/classification models. In IBD, investigators have developed radiomic-based models that can detect inflammation and quantify disease severity better than humans.^6,7^ However, these studies are currently limited to computed tomography and magnetic resonance imaging, and the role of radiomics for IUS has not been investigated. In this study, we aim to assess the feasibility of radiomic analysis of IUS images in IBD and to evaluate if a radiomics-based classification model can accurately differentiate between normal and abnormal IUS images. As a secondary aim, we will compare the radiomics-based performance to a convolutional neural network (**CNN**)-based classification model to understand which method is more effective for extracting meaningful information from IUS images.

## Methods

The study was a single-center, retrospective analysis of adult IBD patients (age ≥18) treated at a tertiary IBD center who underwent IUS during their routine outpatient visit between May 17, 2023, and November 8, 2023. The study was IRB-approved (IRB#3358). For this feasibility study, we focused the analyses on colon images to avoid confounding from imaging differences with the ileum. Images were included if at least a 3 cm colonic bowel wall was visible in the longitudinal axis on the IUS image. Of note, some patients underwent more than one IUS exam during the study period, so images from the same patient but at different time points were also included if the images met the inclusion criteria. For example, IUS images obtained pre- and post-treatment from the same patient could have been included. There were no specific exclusion criteria based on body mass index.

### IUS Protocol and Image Classification

All IUS exams were performed by one IBD specialist who was formally trained by the International Bowel US Group (www.IBUS-group.org) and has performed over 750 IUS exams. Subjects were not required to undergo any fasting, bowel preparation, or ingestion of oral contrast agents prior to the exam. The IUS exams were performed using a GE Logiq e10 using a convex transducer (C2-9 MHz) for global abdominal assessment and a linear transducer (L3–12 MHz) for detailed bowel segment measurements and color Doppler assessment. Each exam followed a consistent standard technique that included a brief survey of the pelvis followed by a complete grayscale and color Doppler evaluation of the colon starting with the sigmoid colon superior to the left iliac vessels in the left lower quadrant of the abdomen until the terminal ileum was identified superior to the right iliac vessels in the right lower quadrant. During these routine exams, standard assessments of the following parameters were obtained and reported for all segments of the bowel (sigmoid, descending, transverse, and ascending colon and terminal ileum) based on international expert consensus^3^: (1) (**BWT**, mm) was measured as the average of 4 measurements, 2 in the longitudinal plane, and 2 in the cross-sectional plane from the lumen-mucosa interface to the muscularis propria-serosal interface, (2) bowel wall hyperemia as measured by the presence or absence of color Doppler signal, with a velocity rate of ±5.2cm/s and graded according the semi-quantitative modified Limberg score (scored 0–3). The presence of inflammatory mesenteric fat, bowel wall echo-stratification, and presence of reactive mesenteric lymph nodes were also evaluated as part of the exam but were not used for the analysis.

For analyses, colon images were classified as either normal or abnormal, with abnormal defined as average BWT > 3 mm or modified Limberg score ≥1. These parameters are most important and sensitive for detecting endoscopically active inflammation.^1^

### Image Post-processing and Radiomics Feature Extraction

**Figure 1.**
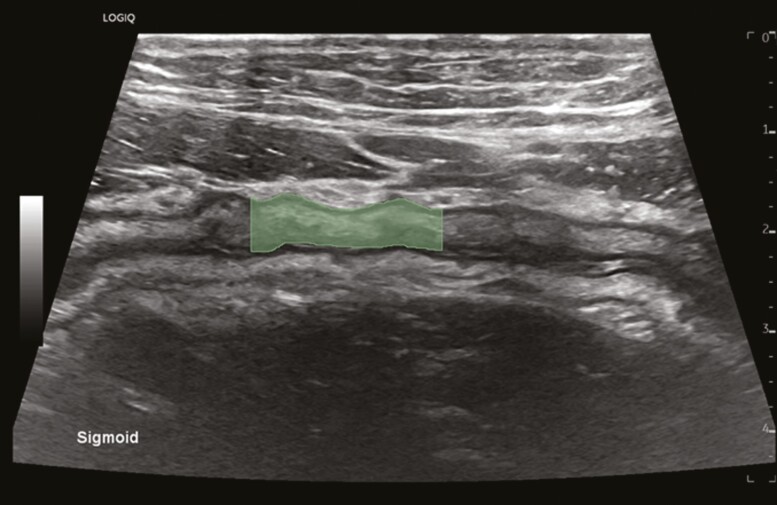
Example of the mask (green) manually drawn on IUS image.

To standardize annotation and reduce bias risk, masks were manually drawn over the bowel wall in the longitudinal axis and were drawn to be 3 cm long with straight edges (Figure 1). All masks were drawn by the IUS expert who performed all the IUS exams. The inner border of the bowel wall was the lumen-mucosa interface, and the outer border of the bowel wall was the submucosa-serosa interface as defined by expert consensus.^3^ Radiomic features were extracted from the original DICOM image and NIFTI segmentation, serving as the region of interest (**ROI**) using Pyradiomics library (v 3.0.1). Pyradiomics was configured with custom settings, including intensity standardization, outlier removal (for standard deviations > 3), and a fixed bin size (binwidth = 25) for gray-level discretization to improve the feature repeatability.^8^ Additionally, to incorporate further information, 4 distinct filtering techniques—wavelet, square root, gradient magnitude, and a Laplacian of Gaussian—were applied to the original ROI.^9^ Wavelet transformation decomposes an image into different frequency components, having 4 sub-bands to represent low/high-frequency information in horizontal/vertical direction. Other filters, such as the square root filter, enhance image contrast, the gradient magnitude highlights edges and boundaries, and the Laplacian of Gaussian detects regions of rapid intensity change.

A total of 858 radiomic features were extracted, comprising both first-order statistics and shape-based metrics, as well as second-order features. The first-order statistics and shape-based metrics provide insights into the distribution of voxel intensities and ROI size. The second-order features encompass the gray-level co-occurrence matrix (**GLCM**), gray-level run-length matrix, gray-level size zone matrix, neighboring gray-tone difference matrix, and gray-level dependence matrix. These second-order features characterize patterns among pixels and voxels within an ROI, considering their spatial arrangement and connectivity. For the subsequent model analysis, we retained 38 features exhibiting linear independence, characterized by a Pearson correlation coefficient ≤ 0.7. These features were further refined using a selection-from-model approach with 5-fold cross-validation, ensuring only those reaching an accumulated importance of 0.95 and appearing in at least 80% of the folds were included.

### Radiomics Feature Analysis

Given the imbalanced nature of our dataset, we utilized balanced bagging with 6 machine learning classifiers as base models for abnormal classification: Logistic regression, Decision Tree, Random Forest (**RF**), Extreme Gradient Boosting (**XGB**), multi-layer perceptron, and k-Nearest Neighbors (**KNN**). **Supplementary Table 3** summarizes each model and their unique strengths and limitations. Because each model has its own learning process and work better for certain data types than others, we used these different models to understand which model best fits our radiomics data. The same features were used in all classifier models. Parameter tuning for each model was performed using grid-search, as outlined in (**Supplementary Table 1**), with an initial focus on optimizing area under the receiver operating characteristic curve (**AUC**) scores via 5-fold cross-validation. We employed a stratified 5-group fold cross-validation, maintaining an 80%/20% train/test ratio to ensure patient-specific data integrity and minimize bias. Subsequently, the optimal parameters derived from this process were applied to the testing dataset. The performance metric for abnormal classification was evaluated using AUC. The reported AUC scores were calculated by averaging the results obtained from evaluations across the different shuffle splits. We utilize xgboost libraries (v 2.0.2) for XGB classifiers and the other 5 classifiers from scikit-learn (v 1.3.0).

### Convolutional Neural Network

We constructed a custom architecture comprising 2 base models (EfficientNet-B1 and EfficientNet-B3) as the backbone of our CNN. this network was trained using both original images and clinical features, such as age, gender, and race as listed in Table 1. All images were consistently cropped to a size of 600 × 300 pixels (**Supplementary Figure 1**) based on the input masks. To reduce the risk of overfitting, we applied a series of transformations through the PyTorch transforms module. These transformations include horizontal flips, color-jittering augmentation strategies, and normalization. Furthermore, we added dropout layers and batch normalization layers for regularization to the CNN model. Our approach involves early stopping, triggered when there is no improvement in the validation set for a specified number of epochs (es_patience), and a scheduled learning rate adjustment (ReduceLROnPlateau). This adjustment dynamically tunes the learning rate during training based on the validation performance.

**Table 1.** Cohort demographics of unique subjects (*n* = 61).

	Total unique subjects (*n* = 61)
Mean age, (years, SD)	40.8 (14.5)
Female, *n* (%)	33 (54.0)
*Race, n (%)*	
White	52 (85.2)
Black	4 (6.6)
Asian	4 (6.6)
Other	1 (1.6)
Prior surgery, *n*(%)	5 (8.2)
Mean disease duration, (months, SD)	155 (116.73)
*IBD diagnosis and phenotype, n (%)*	
Ulcerative colitis	27 (44.3)
Disease location	
Proctitis (E1)	1 (3.7)
Left-sided (E2)	9 (33.3)
Extensive (E3)	17 (63.0)
Crohn’s disease	34 (55.7)
Disease location	
Ileal (L1)	11 (32.4)
Colonic (L2)	11 (32.4)
Ileocolonic (L3)	12 (35.3)
Upper GI (L4)	3 (8.8)
Perianal disease (p)	4 (11.8)
Disease behavior	
Non-stricturing, non-penetrating (B1)	20 (58.8)
Fibrostenosing (B2)	11 (32.4)
Internal penetrating (B3)	3 (8.8)
*Current medications, n (%)*	
Steroids	11 (81.0)
5-aminosalicylate	7 (11.5)
Immunomodulator	3 (4.9)
Biologic/small molecule	45 (73.8)

SD, standard deviation.

During the training phase, we calculated the abnormal class weight to adjust the binary cross-entropy with the logits loss function, addressing imbalanced image classification. Consequently, we employed StratifiedKFold with a parameter (n_splits = 5) for evaluation. The primary performance measures were obtained using AUC scores. Throughout our analysis, we utilized the PyTorch library (v1.13.1 + cu117) as the CNN framework for model training and evaluation.

## Results

**Figure 2.**
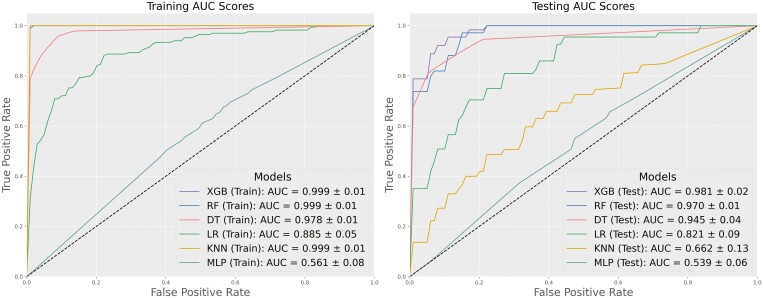
The testing and training receiver operating characteristic (ROC) curves and area under the curve (AUC) scores for 6 different machine learning models: Logistic regression (LR), decision tree (DT), random forest (RF), XGBoost (XGB), K-nearest neighbor (KNN), and multi-layer perceptron (MLP).

**Figure 3.**
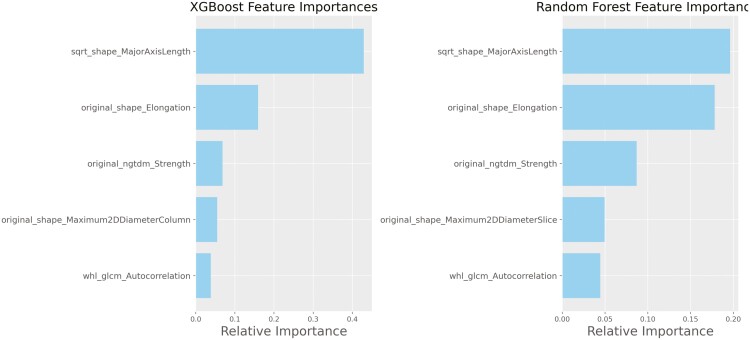
Top 5 feature importance from XG Boost and random forest models.

We analyzed 125 images (33% abnormal) obtained from 61 subjects (Table 1). **Supplementary Table 2** details IUS findings for each unique subject. For this study, 80% of the images were used for training and the remaining 20% for testing. The training and test ROC curves for the 6 classifiers are presented in Figure 2. XGB, RF, and Decision Tree classifiers yielded the best performance for classifying normal and abnormal image with average test AUC 0.981 (95% CI: 0.965, 0.996), 0.970 (95% CI: 0.966, 0.974), and 0.945 (95% CI: 0.909, 0.982), respectively. The XGB classifier model achieved a 5-fold average sensitivity of 0.938, specificity of 0.938, and accuracy of 0.937, respectively (Table 2). RF classifier achieved 5-fold average sensitivity of 0.629, specificity of 0.973, and accuracy of 0.873, respectively. The top 2 performing models, XGB and RF classifiers, identify MajorAxisLength from square root images, Elongation, ngtdm_Strength, and Maximum2DDiameterColumn from original images, along with glcm_Autocorrelation from wavelet images, as the top important features Figure 3. These features reflect the ROI’s axis length, principal component ratio, vertex distance, image primitive intensity (Neighboring Gray Tone Difference Matrix), and texture detail (Gray Level Co-occurrence Matrix). The CNN-based classification model yielded an average training and testing AUC of 0.775 (95% CI: 0.720, 0.830) and 0.754 (95% CI: 0.727, 0.782), respectively.

**Table 2.** Average Accuracy, Sensitivity, and Specificity of the Test Cohort (20% of Images) Using Different Radiomic-Based Machine Learning Classification Models (Calculated Using 5-Fold Cross-Validation and Presented With a 95% Confidence Interval [CI]).

	Accuracy (95% CI)	Sensitivity (95% CI)	Specificity (95% CI)
XG boost	0.937 (0.902, 0.972)	0.938 (0.871, 0.999)	0.938 (0.890, 0.986)
Decision tree	0.897 (0.846, 0.949)	0.938 (0.871, 0.999)	0.878 (0.806, 0.95)
Random forest	0.873 (0.832, 0.914)	0.629 (0.425, 0.834)	0.973 (0.927, 0.999)
Logistic regression	0.834 (0.748, 0.921)	0.683 (0.516, 0.851)	0.882 (0.764, 0.999)
k-nearest neighbor	0.691 (0.652, 0.729)	0.413 (0.293, 0.533)	0.823 (0.762, 0.884)
Multi-layer perceptron	0.659 (0.612, 0.706)	0.283 (0.001, 0.609)	0.812 (0.603, 0.999)

## Discussion

In this feasibility study, we demonstrated that radiomic analysis of IUS images is feasible. We also developed radiomic-based classification models that accurately differentiated between normal and abnormal colon IUS images and performed better than a CNN-based model in our cohort.

AI-based applications have the potential to not only improve workflow efficiency but also the accuracy and standardization of imaging interpretation, leading to greater diagnostic precision. Moreover, in oncology, radiomic-based imaging applications have been developed to predict tumor biology.^10^ With the growth of IUS for IBD, new challenges have emerged. First, performing IUS is operator-dependent and requires expertise, so the steadily increasing number of inexperienced operators performing IUS for IBD increases the risk of diagnostic error. Second, because it is a noninvasive, radiation-sparing, point-of-care tool, IUS offers an ideal research tool for imaging biomarker discovery. However, current approaches are confined to the limited number of IUS parameters used to detect inflammation that have been pre-established by human expert consensus.^3^ AI-based approaches can address these challenges with IUS in IBD. Presently, only one study has explored AI in IUS and developed an automated CNN classification model to detect abnormal bowel segments in Crohn’s disease.^11^ While CNN is excellent for detection and classification with imaging, radiomics allows for a comprehensive analysis of a wide range of features that provide a more detailed and holistic characterization of the underlying biological and pathological processes in the tissue of interest. Radiomics also has the advantage of being able to be integrated with other clinical and “-omic” data for multi-modal analyses to answer a broader spectrum of scientific questions.

To our knowledge, this is the first study to conduct a radiomic analysis of IUS images. Among the different classification models, XGB and RF yielded the top performances for our dataset with XGB achieving the best sensitivity, specificity, and accuracy. These results indicate that XGB is particularly effective for this classification task, potentially making it a preferable choice for similar datasets and objectives. The observed performance advantage of XGB and RF may be attributed to factors such as the data type, sample size, and the specific characteristics of the images used in this study. Our study found that XGB excelled in metrics like AUC, sensitivity, specificity, and accuracy. However, the most effective classification model depends on the data and task specifics. More comprehensive studies are necessary to confirm these results and assess the models’ applicability in various contexts. The superior performance of our radiomics-based model vs. a CNN-based model highlights that radiomics requires less data for model training compared to CNN, which often requires thousands of images. However, as previously mentioned, it is important to remember each approach has different tasks that it is best suited for. Because this is a feasibility study, the differences in model performance provide preliminary data to inform methods for future AI-based IUS studies depending on the desired task. Overall, our findings support the ability of AI to standardize image interpretation and accurately detect inflammation on IUS. Additionally, our study describes a novel imaging analytical method with IUS for IBD that can expand its research capabilities and facilitate future imaging biomarkers discovery studies with IUS. These biomarkers can be used to develop innovative multi-modal prediction models and/or yield new insight into the heterogeneous nature of IBD when combined with clinical and other “-omic” data.

Our study has several limitations. First, our study had a small sample size, so validation of our radiomics model was not possible. Additionally, training a CNN model on a limited sample size risks overfitting. We attempted to mitigate this by applying a series of transformations through the PyTorch transforms module as described above. Nevertheless, the primary aim of this study was to determine the feasibility of radiomics analysis on IUS images, so we planned this initial, modestly sized, proof-of-concept study to inform the design of future larger studies. To ensure the robustness of our ML model, we also used cross-validation, detailed in Table 2, which demonstrates its reliability despite our sample size constraints. These cross-validation metrics robustly indicate our model’s performance, highlighting its validity in our retrospective study’s context. Second, images were obtained and interpreted by a single user, so inter-observer variability could not be assessed. This will be important to evaluate in future studies to determine the generalizability of this approach. Third, masks were manually drawn over the IUS images, which could introduce hidden biases into the input data. We attempted to mitigate the risk of this bias by standardizing how masks were drawn over the IUS images. Fourth, our analysis focused on the colon to establish feasibility, but our study establishes the methods to perform radiomic analyses of the ileum. Finally, we included both UC and CD subjects. While there are no established differences in IUS parameters between CD and UC, there could theoretically be differences on a radiomic level. Determining the feasibility of radiomic analysis on IUS images establishes the foundation for IUS-based radiomic studies focusing on CD or UC or even identifying radiomic differences between the 2 diseases on IUS.

In conclusion, our study not only demonstrated the feasibility of radiomic analysis of IUS images but also developed a radiomic-based classification model that accurately differentiated normal and abnormal IUS images. With these encouraging results, we are working to validate our model in an independent cohort and develop an application for automated bowel wall segmentation to facilitate the scalability of our approach in future studies.

## Supplementary Material

otae034_suppl_Supplementary_Tables_S1-S3_Figure_S1

## Data Availability

Data not publicly available.

## References

[1] Dolinger MT , Kayal M. Intestinal ultrasound as a non-invasive tool to monitor inflammatory bowel disease activity and guide clinical decision making. World J Gastroenterol. 2023;29(15):2272-2282. doi: 10.3748/wjg.v29.i15.2272 37124889 PMC10134421

[2] Bezzio C , Saibeni S, Vernero M, et al. The learning curve for using intestinal ultrasonography. Dig Liver Dis. 2024. doi: 10.1016/j.dld.2024.01.192 38320914

[3] Novak KL , Nylund K, Maaser C, et al. Expert consensus on optimal acquisition and development of the international bowel ultrasound segmental activity score [IBUS-SAS]: a reliability and inter-rater variability study on intestinal ultrasonography in crohn’s disease. J Crohns Colitis. 2021;15(4):609-616. doi: 10.1093/ecco-jcc/jjaa216 33098642 PMC8023841

[4] Gu P , Mendonca O, Carter D, et al. AI-luminating Artificial intelligence in inflammatory bowel diseases: a narrative review on the role of AI in endoscopy, histology, and imaging for IBD. Inflamm Bowel Dis. 2024. doi: 10.1093/ibd/izae030 38452040

[5] van Timmeren JE , Cester D, Tanadini-Lang S, Alkadhi H, Baessler B. Radiomics in medical imaging-“how-to” guide and critical reflection. Insights Imaging. 2020;11(1):91. doi: 10.1186/s13244-020-00887-2 32785796 PMC7423816

[6] Li H , Mo Y, Huang C, et al. An MSCT-based radiomics nomogram combined with clinical factors can identify Crohn’s disease and ulcerative colitis. Ann Transl Med. 2021;9(7):572. doi: 10.21037/atm-21-1023 33987270 PMC8105820

[7] Ding H , Li J, Jiang K, et al. Assessing the inflammatory severity of the terminal ileum in Crohn disease using radiomics based on MRI. BMC Med Imaging. 2022;22(1):118. doi: 10.1186/s12880-022-00844-z 35787255 PMC9254684

[8] Duron L , Savatovsky J, Fournier L, Lecler A. Can we use radiomics in ultrasound imaging? Impact of preprocessing on feature repeatability. Diagn Interv Imaging. 2021;102(11):659-667. doi: 10.1016/j.diii.2021.10.004 34690106

[9] Braghetto A , Marturano F, Paiusco M, Baiesi M, Bettinelli A. Radiomics and deep learning methods for the prediction of 2-year overall survival in LUNG1 dataset. Sci Rep. 2022;12(1):14132. doi: 10.1038/s41598-022-18085-z 35986072 PMC9391464

[10] Limkin EJ , Sun R, Dercle L, et al. Promises and challenges for the implementation of computational medical imaging (radiomics) in oncology. Ann Oncol. 2017;28(6):1191-1206. doi: 10.1093/annonc/mdx034 28168275

[11] Carter D , Albshesh A, Shimon C, et al. Automatized detection of crohn’s disease in intestinal ultrasound using convolutional neural network. Inflamm Bowel Dis. 2023;29(12):1901-1906. doi: 10.1093/ibd/izad014 36794834

