# Supplementary material for: Radiomics-Based Analysis of Intestinal Ultrasound Images for Inflammatory Bowel Disease: A Feasibility Study
Source: Crohns Colitis 360. 2024 May 16;6(2):otae034. doi: 10.1093/crocol/otae034 (PMC11187771; doi:10.1093/crocol/otae034)
Supplement: otae034_suppl_Supplementary_Tables_S1-S3_Figure_S1 [file otae034_suppl_supplementary_tables_s1-s3_figure_s1.docx]

**Supplementary Table 1.** Classifiers and the parameters using in grid-search.

| **Classifier** | **Parameter** |
| --- | --- |
| **Logistic Regression** | C = [0.1, 1.0, 10.0]  penalty='l2' |
| **Decision Tree** | max_depth_values = [1, 2, 3] |
| **Random Forest** | n_estimators = [50, 100]  min_samples_split_values = [0.1, 0.5]  max_depth = [1, 2, 3]  sampling_strategy='all'  replacement=True |
| **eXtreme Gradient Boosting** | min_child_weight_values = [4, 5, 6]  max_depth = [1, 2, 3]  learning_rate_values = [0.1, 0.5] |
| **Multi-Layer Perceptron** | hidden_layer_sizes = [(64,), (64, 32), (64, 64), (64, 128)]  alpha = [100, 0.1, 0.01]  learning_rate_init = [0.001, 0.01] |
| **k-Nearest Neighbors** | n_neighbors = [5, 10, 15]  weights='distance' |

**Supplementary Table 2**. Intestinal ultrasound findings for each subject.

| **Patient #** | **Abnormal Sigmoid** | **Abnormal Descending** | **Abnormal Transverse** | **Abnormal Ascending** | **Abnormal BWT** | **Max BWT location** | **Max BWT (mm)** | **Abnormal hyperemia** | **Max MLS location** | **Max MLS** | **Loss of bowel stratification** | **Inflammatory mesenteric fat** | **Reactive lymphadenopathy** |
| --- | --- | --- | --- | --- | --- | --- | --- | --- | --- | --- | --- | --- | --- |
| 1 | Yes | Yes | No | No | Yes | Sigmoid | 3.9 | Yes | Sigmoid | 2 | No | No | No |
| 2 | Yes | Yes | No | No | Yes | Sigmoid | 4.2 | Yes | Sigmoid | 1 | No | No | No |
| 3 | No | No | No | No | No | Transverse | 2.9 | No | - | - | No | No | No |
| 4 | No | No | No | No | No | Descending | 2.6 | No | - | - | No | No | No |
| 5 | No | No | No | No | No | Sigmoid | 1.7 | No | - | - | No | No | No |
| 6 | No | No | No | No | No | Sigmoid | 2.6 | No | - | - | No | No | No |
| 7 | No | No | No | No | No | Descending | 2.1 | No | - | - | No | No | No |
| 8 | No | No | No | No | No | Transverse | 2.2 | No | - | - | No | No | No |
| 9 | No | No | No | No | No | Ascending | 1.5 | No | - | - | No | No | No |
| 10 | Yes | Yes | No | No | Yes | Descending | 4.3 | Yes | Descending | 2 | Yes | Yes | No |
| 11 | Yes | Yes | No | No | Yes | Sigmoid | 3.6 | Yes | Sigmoid | 2 | No | No | No |
| 12 | Yes | No | No | No | Yes | Sigmoid | 3.4 | No | - | - | No | No | No |
| 13 | No | No | No | No | No | Transverse | 2.6 | No | - | - | No | No | No |
| 14 | Yes | Yes | No | No | Yes | Sigmoid | 3.8 | Yes | Sigmoid | 1 | No | No | No |
| 15 | No | No | No | No | No | Sigmoid | 1.8 | No | - | - | No | No | No |
| 16 | Yes | Yes | Yes | Yes | Yes | Sigmoid | 4.8 | Yes | Sigmoid | 2 | Yes | No | No |
| 17 | Yes | Yes | No | No | Yes | Descending | 4.8 | No | - | - | No | No | No |
| 18 | Yes | No | No | No | Yes | Sigmoid | 5.8 | Yes | Sigmoid | 1 | No | No | No |
| 19 | Yes | Yes | Yes | Yes | Yes | Descending | 7.6 | Yes | Descending | 2 | Yes | Yes | No |
| 20 | No | No | No | No | No | Sigmoid | 1.5 | No | - | - | No | No | No |
| 21 | Yes | Yes | No | No | Yes | Sigmoid | 4.0 | Yes | Sigmoid | 1 | Yes | No | No |
| 22 | No | No | No | No | No | Descending | 1.8 | No | - | - | No | No | No |
| 23 | Yes | No | No | No | No | Sigmoid | 2.9 | Yes | Sigmoid | 1 | No | No | No |
| 24 | No | No | No | No | No | Sigmoid | 1.0 | No | - | - | No | No | No |
| 25 | Yes | Yes | No | No | Yes | Descending | 3.7 | No | - | - | No | No | No |
| 26 | Yes | Yes | No | No | Yes | Sigmoid | 7.7 | Yes | Sigmoid | 1 | No | No | No |
| 27 | No | No | No | No | No | Descending | 1.5 | No | - | - | No | No | No |
| 28 | No | No | No | No | No | Descending | 1.4 | No | - | - | No | No | No |
| 29 | No | No | No | No | No | Ascending | 1.6 | No | - | - | No | No | No |
| 30 | Yes | Yes | No | No | No | Sigmoid | 1.6 | Yes | Sigmoid | 1 | No | No | No |
| 31 | No | No | No | No | No | Sigmoid | 1.4 | No | - | - | No | No | No |
| 32 | No | No | No | No | No | Descending | 0.7 | No | - | - | No | No | No |
| 33 | No | No | No | No | No | Sigmoid | 3.0 | No | - | - | No | No | No |
| 34 | Yes | Yes | No | No | Yes | Descending | 4.6 | Yes | Descending | 1 | Yes | Yes | No |
| 35 | No | No | No | No | No | Sigmoid | 1.5 | No | - | - | No | No | No |
| 36 | No | No | No | No | No | Sigmoid | 2.8 | No | - | - | No | No | No |
| 37 | Yes | No | No | No | Yes | Sigmoid | 4.2 | Yes | Sigmoid | 1 | No | No | No |
| 38 | Yes | Yes | Yes | No | Yes | Descending | 4.0 | Yes | Descending | 1 | Yes | No | No |
| 39 | No | No | No | No | No | Descending | 1.6 | No | - | - | No | No | No |
| 40 | Yes | Yes | No | No | Yes | Descending | 5.3 | Yes | Descending | 2 | No | Yes | No |
| 41 | No | No | No | No | No | Descending | 2.8 | No | - | - | No | No | No |
| 42 | No | No | No | No | No | Descending | 1.7 | No | - | - | No | No | No |
| 43 | No | No | No | No | No | Transverse | 2.4 | No | - | - | No | No | No |
| 44 | Yes | Yes | No | No | Yes | Descending | 4.6 | No | - | - | No | No | No |
| 45 | No | No | No | No | No | Sigmoid | 2.5 | No | - | - | No | No | No |
| 46 | Yes | No | Yes | No | Yes | Transverse | 3.4 | No | - | - | No | No | No |
| 47 | No | No | No | No | No | Sigmoid | 2.4 | No | - | - | No | No | No |
| 48 | Yes | Yes | No | No | Yes | Descending | 3.9 | Yes | Sigmoid | 1 | No | No | No |
| 49 | No | No | No | No | No | Descending | 2.4 | No | - | - | No | No | No |
| 50 | No | No | No | No | No | Transverse | 2.1 | No | - | - | No | No | No |
| 51 | No | No | No | No | No | Sigmoid | 2.0 | No | - | - | No | No | No |
| 52 | No | No | No | No | No | Sigmoid | 1.6 | No | - | - | No | No | No |
| 53 | No | No | No | No | No | Descending | 1.8 | No | - | - | No | No | No |
| 54 | No | No | No | No | No | Sigmoid | 2.9 | No | - | - | No | No | No |
| 55 | No | Yes | N/A | N/A | No | Descending | 2.1 | Yes | Descending | 1 | No | No | No |
| 56 | No | No | No | No | No | Sigmoid | 2.5 | No | - | - | No | No | No |
| 57 | No | No | No | No | No | Descending | 2.4 | No | - | - | No | No | No |
| 58 | No | No | No | Yes | Yes | Ascending | 4.1 | No | - | - | No | No | No |
| 59 | No | No | No | No | No | Sigmoid | 2.1 | No | - | - | No | No | No |
| 60 | Yes | Yes | No | No | Yes | Sigmoid | 4.2 | No | - | - | No | No | No |
| 61 | Yes | Yes | No | No | Yes | Descending | 6.9 | Yes | Descending | 3 | Yes | Yes | No |

BWT, bowel wall thickness; MLS, modified Limberg score

**
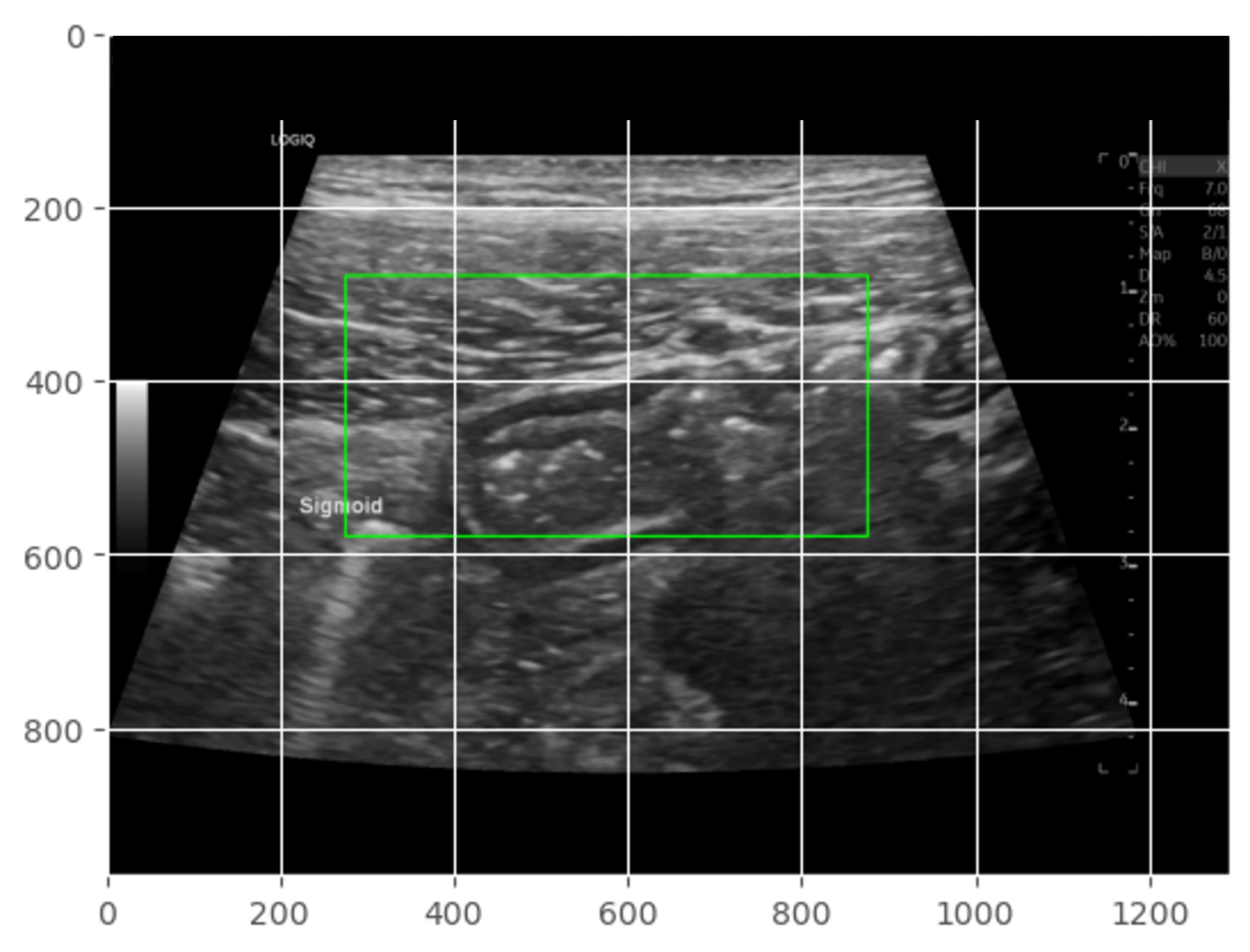
Supplementary Figure 1.** Example cropped image for CNN model.

**Supplementary Table 3.** Summary of definition, strengths, and limitations of each machine-learning classifier models used in the study.

| Model | Definition | Strengths | Limitations |
| --- | --- | --- | --- |
| Logistic Regression | Classification model that predicts the probability of a binary outcome based on one or more predictor variables. | - Provides probabilities for outcomes. - Coefficients offer insights into feature importance. | - Assumes a linear relationship between features and outcome. - Limited flexibility for capturing complex patterns in data. - Sensitive to outliers. - Perform may be impacted by highly correlated features. |
| Decision Tree | Non-linear model that divides data into subsets based on the values of input features, forming a tree-like structure where each internal node represents a decision based on a feature, and each leaf node represents the outcome. | - Easy to interpret. - Handles both numerical and categorical data. - Automatically selects important features. - Non-parametric. | - Prone to overfitting - Can be sensitive to small data variations. - Small changes in data can result in different trees (instability). - Difficulty in capturing relationships between features that require interactions. |
| Random Forest | An ensemble learning method that constructs multiple decision trees during training and outputs the mode of the classes (classification) or the average prediction (regression) of the individual trees. | - Reduces overfitting by averaging multiple decision trees. - Good for high-dimensional data. - Robust to outliers and noisy data. - Provides feature importance estimation. | - More difficult to interpret compared to individual decision trees. - Potentially poor performance with very imbalanced datasets. |
| eXtreme Gradient Boosting | An ensemble learning method that builds multiple decision trees sequentially, each tree correcting the errors of the previous one, thereby improving predictive accuracy. | - Highly accurate (frequently wins machine learning competitions). - Handles missing data well. - Regularization techniques reduce overfitting. - Provides feature importance estimation. | - Requires careful tuning of hyperparameters. - Prone to overfitting if hyperparameters are not tuned properly. - Less interpretable compared individual decision trees. - May not perform well with noisy or irrelevant features. |
| Multi-Layer Perceptron | A neural network model composed of multiple layers of nodes with each layer fully connected to the next one. It can learn complex patterns in data through non-linear transformations. | - Can learning complex patterns in data. - Good for large datasets. - Can learn non-linear relationships between features and target. - Robust to irrelevant features. | - Requires careful tuning of hyperparameters. - Sensitive to feature scaling. - Prone to overfitting, especially with insufficient training data. - Less interpretable than simpler models like individual decision trees. |
| k-Nearest Neighbors | A non-parametric method used for classification, where the output is based on the majority vote (classification) or the average (regression) of the k nearest data points in the feature space. | - Easy to interpret. - No training required; new data can be inputted without retraining. - Good for small datasets. | - Requires careful selection of the number of neighbors (k) and distance metric. - Sensitive to the scale of features. - Not ideal for high-dimensional data. - Not good with imbalanced datasets. |
